# Managing a *Salmonella* Bredeney Outbreak on an Italian Dairy Farm

**DOI:** 10.3390/ani14192775

**Published:** 2024-09-26

**Authors:** Camilla Torreggiani, Cosimo Paladini, Marcello Cannistrà, Benedetta Botti, Alice Prosperi, Chiara Chiapponi, Laura Soliani, Ada Mescoli, Andrea Luppi

**Affiliations:** 1 Istituto Zooprofilattico Sperimentale Della Lombardia e Dell’Emilia-Romagna (IZSLER), “Bruno Ubertini”, 25124 Brescia, Italy; 2 Local Health Authority of Parma, 43100 Parma, Italy; 3 Veterinary Practitioner, 43100 Parma, Italy

**Keywords:** dairy farm, outbreak management, *Salmonella*

## Abstract

**Simple Summary:**

*Salmonella enterica* subsp. *enterica* infections critically affect cattle health, producing high rates of morbidity and mortality in calves and reducing the performance of adult cows. Some European countries implemented surveillance and control programs decades ago, but in Italy, there is no national control program for dairy cows. This study describes an outbreak of *Salmonella* Bredeney in a dairy herd and the consequent control strategies applied. Control strategies included sample collection visits to the farm in order to identify the prevalence of *Salmonella* spp. and assess biosecurity levels. In this outbreak, there was a higher prevalence in calves and this condition was associated with poor biosecurity practices. Subsequently, the practitioner and farmer were provided with a health management plan to reduce the prevalence and control the outbreak. During the follow-up period, monitoring and testing revealed repeated negative results, indicating adequate control over the outbreak. The procedures undertaken in this project made it possible to collect useful data for the definition of measures for the management of outbreaks of salmonellosis in dairy cows.

**Abstract:**

Salmonellosis in dairy cattle represents an increasing problem for both animal and public health. Nevertheless, in Italy, there is no control plan in place on dairy farms. The aim of this study was to describe a *Salmonella* Bredeney outbreak that occurred on a dairy farm and the measures that were adopted to control the outbreak. Management consisted in identifying the spread of infection and assessing the environmental contamination of *Salmonella* spp. and the associated risk factors. After the farm visit, laboratory investigations showed that 48% of rectal swabs collected from calves and 33% of environmental samples were positive for *S*. Bredeney, and a poor biosecurity level was detected. The farmer and practitioner were provided with a health management plan to control the spread of *Salmonella* spp., followed by a monitoring period and a follow-up visit in which all samples resulted negative. The results demonstrated the efficacy of indirect prophylaxis measures in reducing the circulation of *Salmonella* spp., leading to the extinction of the outbreak. Collaboration with farmers, practitioners, and public health veterinarians and the introduction of measures reported in the health management plan constitute a possible model for the management of *Salmonella* spp. outbreaks in dairy herds, even in complex farm situations.

## 1. Introduction

*Salmonella enterica* subsp. *enterica* infections pose a threat to the dairy cattle industry due to economic losses in infected herds, mainly because of the costs of clinical disease, which include diagnostic laboratory investigations and treatment of clinical cases. Cleaning and disinfection, as well as control and prevention measures, pose additional costs [1]. *Salmonella* Dublin, *S*. Typhimurium, and the *S*. Typhimurium monophasic variant 1,4,[5],12:i:- are generally associated with salmonellosis in calves and adult cows, causing mild to severe illness [1]. In addition, other, less frequently detected serotypes must be considered relevant to cattle farms. Clinical signs of bovine salmonellosis may include diarrhea, fever, anorexia, and dehydration, while in milking animals, milk production severely drops and abortions may also occur [2]. Infected animals can become shedders and spread the organism for a varying period of time, as well as intermittently in the environment, after either clinical or subclinical infections. In favorable conditions, *Salmonella* spp. can survive in the environment outside the host for a long time [3,4]. Transmission between animals is fecal–oral, and the contamination of the environment, feed, and water play an important role in the epidemiology of salmonellosis [3]. Herd management is a crucial factor; free stalls compared with tie-stall housing systems have been described to increase contact between animals, and in larger herds, cows and calves may be more densely housed, which could promote the spread of infections and increase environmental contamination. Some European countries have implemented national or regional surveillance, control, or eradication programs [5], while in Italy, there is no national control program for salmonellosis in cattle. Thus, data on the occurrence of *Salmonella* spp. in cows are collected in the framework of Directive 2003/99/EC [6]. Nevertheless, clinical salmonellosis is a notifiable disease in Italy. Considering the increasing importance of salmonellosis in cattle and its potential impact not only on animals but also on public health, it is of pivotal importance to optimize the data collection system, as well as to standardize the methods used for epidemiological investigations in case of outbreaks [7].

These considerations led to the need to find strategies to manage *Salmonella* outbreaks. A research project funded by the Istituto Zooprofilattico Sperimen tale della Lombardia e dell’Emilia-Romagna (IZSLER), AUTOFIN_SALMONEL_BOV “Drafting a manual for the management of salmonellosis outbreaks in dairy farms”, focused on managing S*almonella* outbreaks on dairy farms in the Emilia-Romagna region. The present study describes an outbreak of *S.* Bredeney in a dairy herd and the consequent control strategies applied.

## 2. Materials and Methods

### 2.1. Farm Characteristics

This case report involved an intensive, loose-housing dairy farm for the production of Parmigiano-Reggiano cheese with a total of 600 heads of Holstein Friesian cattle, with a year-round calving pattern. Milking cows (300) had an average daily production of 34 kg of milk/cow. The average productive lifespan of the cows was 2.4 lactations. The most frequent reasons for culling were aging, mastitis, and lameness, with an average of 35% cows culled. Artificial insemination procedures were performed. The resting area type for lactating cows consisted of cubicles with straw bedding materials. Dry cows also had an external paddock.

Colostrum was stored in a colostrum bank and valued by using a Brix refractometer. On this farm, calves were usually fed 4 L of colostrum only once after birth.

For the first two months of life, calves were housed in single boxes, while older calves were housed in boxes with straw bedding material with other calves of the same age. After collection and storage, slurry was deposited on pastures. No animals had been purchased in the last few years. Barns were naturally ventilated, while milking cows’ barns were ventilated by fans. In addition, there were misting fans in the milking parlor and waiting area. Natural light was sufficient during the day, and lighting control was available during the night, combined with an 8 h dark period. The farm used 60% home-grown hay and grass for their livestock, without using silage or fermented forage. Drinking well water was provided in a livestock water trough and was analyzed once a year. Straw bedding materials were changed once a month, manure scrapers were used twice a day, and straw bedding was added to calves’ cages every other day and cleaned using high pressure washers when calves were moved to the post-weaning youngstock boxes. Rodent and insect control measures were performed, while there were no control measures for wild birds or synanthropic animals. Antimicrobial treatments data showed that antibiotic use for the year 2020 was 6.32 Defined Daily Dose Animal for Italy (DDAit), higher than the national median (2.56 DDAit).

The vaccination schedule included a live attenuated intranasal bovine parainfluenza 3 virus (PI3) and bovine respiratory syncytial virus (BRSV) vaccine in calves; inactivated vaccine for bovine coronavirus (BCoV), bovine rotavirus (BRV), and *Escherichia coli* administered intramuscularly in dry cows; infectious bovine rhinotracheitis (IBR) marker vaccine in milking cows; and inactivated bovine viral diarrhea virus (BVDV), inactivated PI3 vaccine, and live BVRS vaccine in milking cows.

### 2.2. Outbreak Description

In July 2021, two deceased 7-day-old calves were sent to the Diagnostic Laboratory of Parma (IZSLER) for post-mortem examination and laboratory investigations. The practitioner reported that the calves initially showed watery diarrhea and hematochezia, followed by weakness, recumbency, severe dehydration, and death. No enteric disorders, abortions, or other significant clinical signs were observed in cows. Necropsy, performed on both calves using standardized procedures, showed severe dehydration, sunken eyes, and yellowish, blood-stained, foul-smelling scour around the perineal region and tail. Gross lesions observed were hepatomegaly, enlarged spleen, catarrhal to catarrhal–hemorrhagic enteritis, catarrhal abomasitis, mesenteric lymphadenitis, and pulmonary hemorrhages. Bacteriology was performed from the liver, kidney, spleen, intestinal content, lymph nodes, and lungs of each calf. Briefly, samples from the tissues reported above were plated on blood agar and on Gassner agar (a selective medium for *Enterobatteriaceae*). The latter allows for the differentiation of lactose-fermenting (such as *E. coli*) and lactose-non-fermenting Gram-negative enteric bacilli. Plates were incubated aerobically overnight at 37 °C. Subsequently, lactose-negative colonies were further plated on Xylose Lysine Deoxycholate agar (XLD) (Oxoid Holding Ltd., Hants, UK) and Brilliant Green Agar (BGA) (Biolife S.R.L., Milano, Italy) and incubated overnight at 37 °C [8]. Colonies with morphology characteristic of *Salmonella* spp. were typed with biochemical and serological methods. Serotyping by serological rapid agglutination techniques, through the characterization of somatic, flagellar and capsular antigens, was performed according to ISO/TR 6579-3:2014 [8,9,10]. Final serological characterization was performed according to the scheme of Kauffmann–White–Le Minor [11]. Bacteriological investigations led to the isolation of *S.* Bredeney from all samples collected from both calves.

Antimicrobial susceptibility testing was performed on the strain isolated from tissue samples collected during the necropsies, by the Kirby–Bauer disk diffusion method, using a panel of 10 antimicrobials: nalidixic acid (30 µg), amoxicillin and clavulanic acid (20–10 µg), ampicillin (10 µg), cefazolin (30 µg), enrofloxacin (5 µg), florfenicol (30 µg), gentamicin (10 µg), kanamycin (30 µg), tetracycline (30 µg), and trimethoprim + sulfamethoxazole (1.25–23.75 µg). The *Salmonella* spp. isolate was classified as susceptible, intermediate, and resistant, following CLSI interpretative criteria [12]. The *Salmonella* Bredeney strain tested was resistant to kanamycin and tetracycline, but it was susceptible to all the other antimicrobials tested.

Investigations for BCoV [13], BRV [13], and BVDV [14] were performed by PCR. The samples were positive for bovine coronavirus, while BRV and BVDV were negative.

### 2.3. Outbreak Management

Outbreak management consisted of four main steps. In the first step, a farm visit was conducted in order to perform an epidemiological investigation and to assess the welfare and biosecurity levels of the herd, the prevalence of *S.* Bredeney, and the presence of other *Salmonella* serotypes. The epidemiological investigation, as well as welfare and biosecurity levels, were assessed using a checklist for the control of *paratuberculosis* and *Salmonella* Dublin [15], and a risk scoring form and graphics were used to visualize the high-risk areas in the herd and to evaluate transmission pathways. These spread-sheets are appendices to a Manual for Advisors in Denmark. The risk scoring form was used to go through the herd systematically and assign risk scores to different relevant barn sections and management practices. The maximum scores were decided by the authors of the manual according to existing knowledge about risk factors for the spread of *S.* Dublin in cattle herds, and they were weighted so that the most critical areas for control counted most in the total sum of risk scores [16,17].

The prevalence of *Salmonella* spp. was assessed by collecting rectal swabs from all calves (from birth to weaning age), boot swabs from youngstock’s, dry cows’, and milking cows’ boxes, and sponge sticks on manure scrapers (Table 1). All these samples were tested using bacteriological techniques that included an enrichment step [8], and bulk milk samples were tested using real-time PCR [18].

In the second step, a health management plan (HMP) was drafted to summarize and evaluate the risk scores and to plan actions based on the results of the epidemiological investigations carried out and analytical results obtained in step 1. The HMP was made up of a set of measures that consider the characteristics of the farm, such as its structural and hygienic elements. The HMP, implemented and shared with both the farmer and the practitioner, was a tool to increase the hygiene of livestock farming and to improve its health and welfare aspects with direct and indirect prophylaxis measures, aiming to eliminate or to strongly reduce salmonellosis cases.

In the third step, the measures contained in the HMP were applied, which first consisted of a deep cleaning protocol.

The fourth step was characterized by a monitoring period, during which individual samples were collected by the public health authority from calves that showed clinical signs, from cows 3 weeks before parturition, and from all 2-day-old calves to detect infected animals and to rule out chronically infected animals. Six months after the end of the monitoring period, a follow-up was performed by a farm visit repeating the same environmental sampling performed during the first farm visit and reported in Table 1 (calving cow area, dry cow area, and youngstock area) and to re-evaluate management practices with the checklist previously used.

## 3. Results

### 3.1. First Step

After the farm visit, the laboratory investigations showed that 48% of rectal swabs collected from calves were positive for *S*. Bredeney, and only six of them belonged to diarrheic calves, all of them under 14 days of age. Five out of fifteen (33%) environmental samples collected from the milking cows’ group, dry cows’ group, and youngstock with sponges on automatic passageway scrapings, boot swabs, and pooled samples of feces were positive for *S*. Bredeney. Bulk tank milk analysis was negative (Table 2).

**Figure 1:**
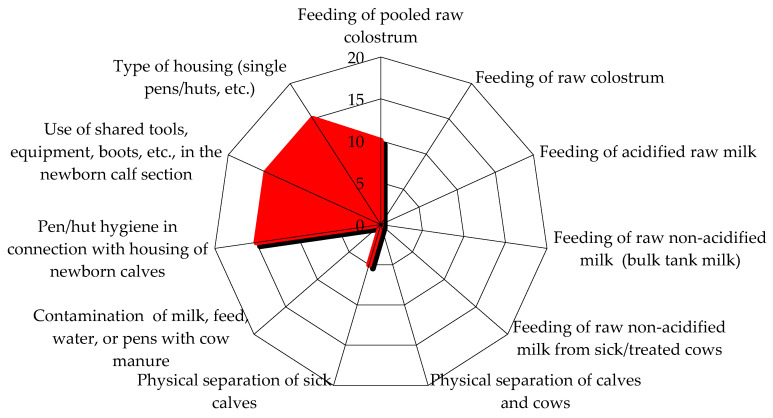
Risk score graphic for pre-weaning calves’ area on the first visit. The graphic highlights elements that score a higher risk; the higher the risk score, the more extended the highlighted area is. The items pen hygiene in connection with housing of newborn calves, the use of shared tools, equipment, boots, etc., in the newborn calf section, and the type of housing showed the highest risk.

**Figure 2:**
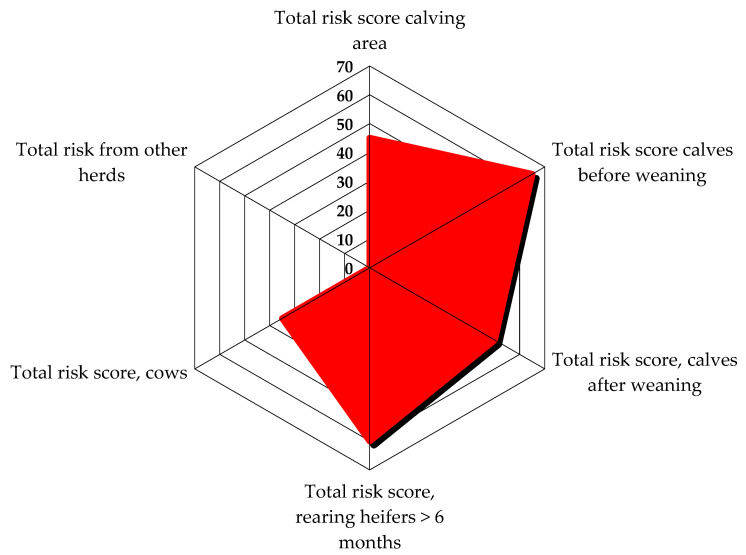
Total risk score in areas of the herd on the first visit. The graphic highlights elements that score a higher risk; the higher the risk score, the more extended the highlighted area is. Calves before weaning show the highest risk, followed by heifers (>6 months) and calves after weaning.

The epidemiological investigation on the first visit highlighted scarce biosecurity levels in the herd. In particular, external biosecurity analysis showed that the main critical aspects revealed by the checklist were the absence of a vehicle disinfection system at the entrance to the herd and the rendering service for the final removal of carcasses, which usually passed through the herd, near the cows’ housing areas. Internal biosecurity evaluation showed that the all-in all-out principle was not respected, overcrowding was observed in the milking cows’ and dry cows’ area, bedding material removal and replacement was on a monthly basis, the same boots and clothes were used in different barns, and no quarantine box was available for every category of animals; also, synanthropic animals such as cats, pigeons, starlings, and hens were present in the herd. In addition, there was an external paddock for dry cows, so contact with wild animals was possible. Figure 1 and Figure 2 show the risk score graphics from the epidemiological investigation checklist. The first figure reports a risk score graphic for the pre-weaning calves’ area, as pre-weaned calves were the main category involved in the outbreak; the second figure shows the total risk score for the entire area of the herd and possible risks from other herds (Figure 1 and Figure 2).

### 3.2. Second and Third Steps

After the first visit, the HMP was drafted as reported above in order to find strategies to reduce the prevalence of *S.* Bredeney in the herd and also to suggest ways to improve the biosecurity levels and the hygiene parameters, in particular on passageways scrapers and housing areas, to reduce the contamination of dried fecal material. The cleaning protocol was planned to include descaling and removal of all organic material from the structures, in particular the calves’ pens, calving maternity pen, and manure scrapers in the milking cows’ and dry cows’ area. All structures, means of transport used for livestock, and tools used in livestock farming suspected to be contaminated had to be adequately cleaned, so it was suggested that movable tools, wood, ropes, and all organic material be removed before applying the disinfectant.

The suggested cleaning protocol consisted in the following procedure: scraping and removing coarse dirt mechanically (with the aid of brooms, vacuum cleaners, blowers, etc.); initial rinsing with hot water at a temperature higher than 45 °C, but lower than 60 °C, to dissolve fats and facilitate their detachment; then, applying the detergent in order to loosen dirt from the surfaces and allow it to move away with the next rinse. Subsequently, the protocol included rinsing with water at tap temperature, then applying the disinfectant after drying the surfaces, according to the dilution and prescribed methods of use; leaving the disinfectant to act for at least 6 h; and finally, rinsing with water at tap temperature, preparing bedding on dry surfaces and reintroducing the animals. The same treatment was suggested for feeders, drinkers, calf milk buckets, teats for calves, cages, etc.

The cleaning protocol also involved a workflow which suggested that operators of the calving area began cleaning procedures with healthy animals and subsequently moved on to infected animals. High-pressure washing was not recommended due to the risk of environmental cross-contamination and the aerosolization of contaminated material, which can increase the risk of infection in workers and animals. High-pressure washers effectively remove coarse material, such as manure, but they are not effective in eliminating bacterial biofilms. A list of effective disinfectants was also suggested. The disinfectant used on this farm was quaternary salts of ammonium because they are effective on Gram-negative bacteria such as *Salmonella* spp. and were already available on the farm.

It was also recommended to keep positive animals separated from the others, so it was important to define an area as a hospital pen. The use of disposable boots and gloves for different areas was prescribed. Disposable gloves were used in the calves’ pen during daily cleaning procedures and a boot sanitization point was implemented at the entrance to the calving area, because the use of dedicated boots or use of disposable shoes/overshoes was not feasible for operators.

The entrance of synanthropic and wild animals was forbidden. In addition, corrective measures were adopted, such as the improvement of routine hygiene procedures, implementation of an appropriate pest control system, systematic cleaning and disinfection of new-born calves’ cages, and proper disinfection of the equipment used to feed calves (Table 3).

Male calves were usually sold to other finishing farms, so to control the spread of infection to veal calf farms, all new-born calves were sampled at least twice within 7 days, and the movement of positive calves was forbidden. According to the HMP, positive clinically affected animals were isolated and treated with antimicrobials. Animal movement or reintroduction was scheduled only after at least two negative bacteriological tests on rectal swabs, the first carried out 3 days after the end of treatments, the second 5–7 days after the first negative sample. In case of repeated positive results, it was advised that animals be culled. It was suggested that asymptomatic animals be isolated and avoid antimicrobial treatment. Animal movement was allowed only after at least two negative tests, as mentioned above [19].

In Italy, there are no commercial vaccines available for *Salmonella* spp. in cattle, so in cases where vaccination is considered to be a control tool, a *Salmonella*-inactivated autogenous vaccine would be necessary. However, in this case, vaccination was not adopted during this outbreak, as the farmer, in agreement with the practitioner, was not willing to add a vaccination to the vaccination plan.

### 3.3. Fourth Step

**Figure 3:**
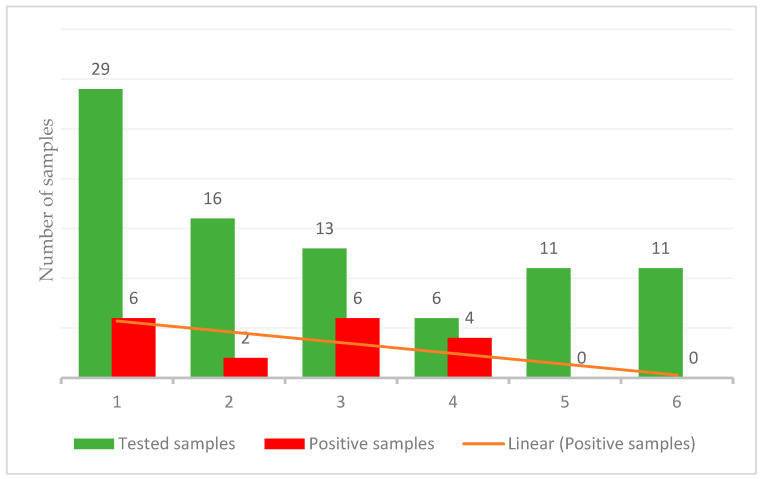
Results of rectal swab sampling during the monitoring period. A total of 18 out of 86 (21%) rectal swabs collected from calves were positive for *S*. Bredeney. Dates of sampling: 1. (6 September); 2. (27 September); 3. (6 October); 4. (12 October); 5. (29 October); 6. (10 November).

**Figure 4:**
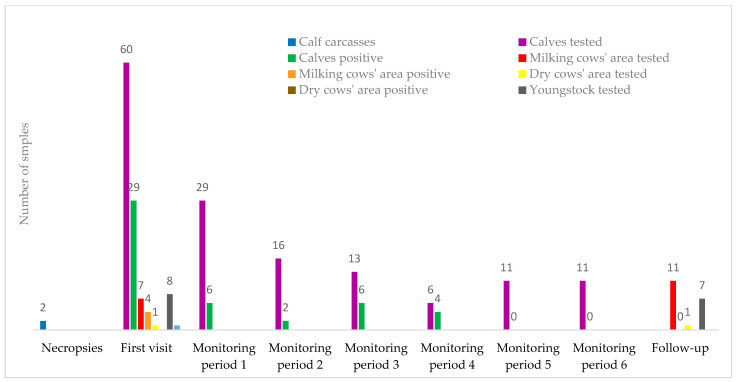
Overview of the outbreak from the beginning (July 2021) through the first visit (August 2021), monitoring period, and follow-up (end of November 2021). Dates of sampling: 1. (6 September 2021); 2. (27 September 2021); 3. (6 October 2021); 4. (12 October 2021); 5. (29 October 2021); 6. (10 November 2021).

During the monitoring period, from September to November 2021, 21% of rectal swabs collected from calves were positive for *S*. Bredeney (Figure 3 and Figure 4).

**Figure 5:**
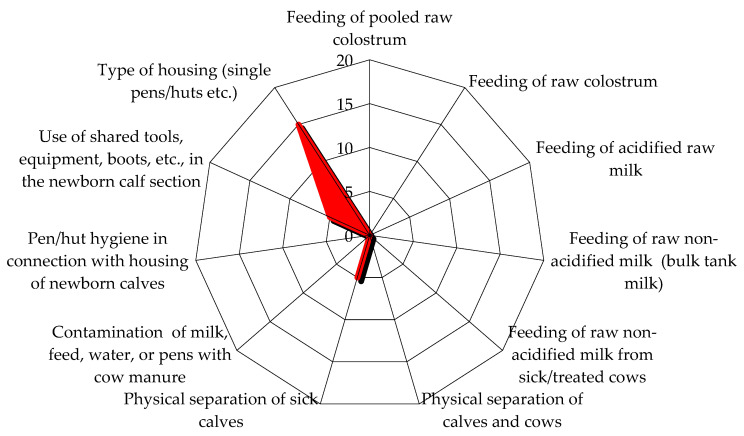
Risk score graphic for pre-weaning calves’ area at follow-up. The graphic highlights elements that score a higher risk; the higher the risk score, the more extended the highlighted area is. The graphic describes a reduction in risk scores compared with the first visit (Figure 1). The item type of housing scores the highest risk, as newborn calves are housed individually or in pairs in pens where the calves are separated by bars and are therefore not physically separated. The use of shared tools shows an improvement compared to the first visit, as only frequently cleaned, dedicated tools are currently used in the newborn calf section.

**Figure 6:**
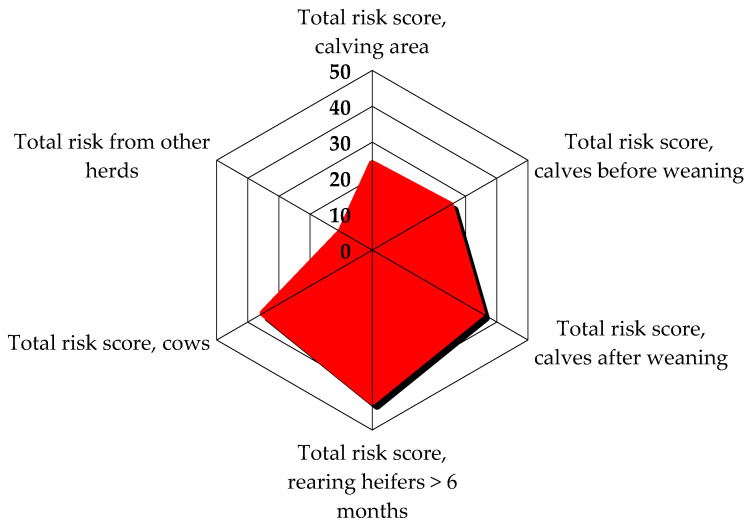
Total risk score in areas of the herd at follow-up. The graphic highlights elements that score a higher risk; the higher the risk score, the more extended the highlighted area is. In this graphic, calves before weaning area show a lower risk score compared with the first visit (Figure 2).

After two negative results were obtained on all samples collected at the end of October and at the beginning of November 2021, the monitoring period was interrupted. All the environmental samples collected six months after the end of the monitoring period (follow-up visit) were negative and new cases of salmonellosis were not observed. Figure 5 and Figure 6 show that risk scores were reduced in comparison with the first visit, especially for pre-weaning calves. The total score for calves after weaning and heifers remained the same because the structural risk elements of the herd could not be improved. The results obtained, together with the absence of clinical signs of salmonellosis and the adoption of the HMP by the practitioner and farmer, led to the revocation of further control measures.

## 4. Discussion

Salmonellosis is a significant disease in animals, both due to the possible health and economic impact and due to the public health implications resulting from the zoonotic nature of the infection [20,21]. Possible forms of *Salmonella* spp. infections are enteric, septicemic, and reproductive. Although reproductive losses are only of concern in adult cows, enteric disease can be seen at any age, from newborn calves through to adulthood [22,23].

Bovine salmonellosis can be caused by different serotypes; however, the most frequent, responsible for the majority of reported cases, are *S.* Typhimurium, including its monophasic variant *S.* 1,4,[5],12:i:-, and *S.* Dublin, a host-adapted serotype [24,25].

In fact, as reported by the 2018 Entervet report [26], the most frequently isolated serotypes in cattle are *S.* Typhimurium and its monophasic variant, identified in 42.6% of the total isolates from cattle in 2018, followed by *S.* Dublin, with an incidence of 31.8%.

A retrospective study of *S. enterica* strains submitted to the Wisconsin Veterinary Diagnostic Laboratory from 2006 to 2015 showed that among 5000 isolates detected, *S.* Dublin was the most prevalent serotype identified, accounting for a total of 1153 isolates (23% of the total) [27]. Along with *S.* Dublin, *S.* Cerro (16%), *S.* Newport (14%), *S.* Montevideo (8%), *S.* Kentucky (8%), and *S.* Typhimurium (4%) comprised the top six most isolated serotypes. In a comprehensive study from the north-eastern United States in 2009, more than 800 dairy herds were involved. The most frequently detected serotype was *S.* Newport, accounting for 41% of cases, followed by *S.* Typhimurium, accounting for almost 20% of cases [28]. Even though *S*. Bredeney is not frequently detected in cattle, it was relevant in this case. In fact, *S.* Bredeney is sporadically identified in cattle but, as in this case, can represent a relevant serotype, able to bring about disease and an important economic impact [29]. There are few publications regarding *S*. Bredeney in cattle, but in 1994, Marley et al. [30] reported that *S.* Bredeney infections in cows had frequently been reported in France. These authors described an outbreak on a farm comprising 160 milking cows. During the first 2 months of the outbreak, cases of fever (68% of cows), dysentery (80%), and abortions (6.9%) were associated with *Salmonella* isolations in feces or in products of abortion. *S.* Bredeney was recovered from the milk tank, but individual milk sampling was not practicable. Authors report that in that specific outbreak, in several respects, the results were similar to those observed with *S*. Dublin, a serotype considered to be primarily adapted to cattle. Cormincan et al. [29] reported that *S.* Bredeney is a well-recognized serotype isolated from poultry, other animals, and the environment, and that it is an uncommon human pathogen associated with occasional outbreaks. The authors also added that *S.* Bredeney had emerged as the third most identified serotype among human clinical isolates, with reference to the Irish National Salmonella Reference Laboratory in the years 1998 to 2000. In 1998, Baker and colleagues [31] reported that *S*. Bredeney had been frequently isolated from a range of food products, animal sources, water, and sewage effluent in the period 1988–1993.

In the outbreak reported in this study, bulk tank milk analysis was negative, and sampling was conducted by the local public health authority due to the destination of the milk produced and the possible associated risks. In this regard, it is important to highlight that in the production of long-seasoned cheeses (Grana Padano and Parmigiano-Reggiano), the high cooking temperatures (55 °C), salting, acidification of the curd carried out by lactic bacteria, and long seasoning lead to the complete inactivation of *Salmonella* spp. [32]. Therefore, the risk of salmonellosis linked to the consumption of this type of dairy products appears negligible [32].

In the outbreak described in the present study, different areas of the farm and a high number of samples from calves tested positive, which made huge impact on the herd. Also, the management of the outbreak lasted a few months.

The most important risk factors observed on the farm were the frequency of cleaning, the presence of synanthropic animals, and the misuse of protective clothing. Overcrowding and poor environmental hygiene, as observed in both milking cows’ and dry cows’ areas, were considered predisposing factors for the spread of the infection. Improving cleaning and disinfection procedures during the outbreak and during the monitoring period was considered a key point to reduce the spread of *Salmonella* within the herd. A specific protocol of workflow and hygiene, as suggested in the HMP, should be considered an important measure to reduce the incidence of clinical cases, especially in the calving area. In this study, indirect prophylaxis, thereby implementing internal biosecurity, can be considered crucial to gradually reducing cases of salmonellosis on the farm. In most cases, there are multiple factors involved in precipitating the *Salmonella* outbreak and in order to be successful, health management plans have to be designed based on specific characteristic of the farm, as considering different aspects of the outbreak and the serotype involved are of critical relevance. For instance, the host-adapted serotype *S*. Dublin has the specific ability to produce long term carriers that shed bacteria in the environment, therefore becoming a steady source of infection [33,34]. In our study, the serotype involved was *S*. Bredeney, a non-host-adapted serotype that is rarely detected in cattle; therefore, our health management plan focused more on increasing levels of biosecurity and disinfection practices.

The activities described in this case report, such as the production of an HMP and the strategic measures contained therein, allowed for the definition of targeted and joint actions with the involvement of different professional figures such as public health veterinarians, the practitioner, and the farmers. The actions undertaken made it possible to obtain an improvement in some critical points present on the farm and to contribute to the management of the salmonellosis outbreak described.

## 5. Conclusions

Considering the increasing importance of salmonellosis in cattle and its potential impact not only on animals but also on public health, it is critically important to collect data and to use standardized methods and approaches for epidemiological investigations in case of an outbreak. *S.* Bredeney is more common in poultry, but this study highlighted that this serotype can be detected on dairy farms and have a severe economic impact on the herd.

The measures applied in this outbreak were designed to limit the spread of the infection within the herd and between farms, as well as the transmission to humans, in a One Health perspective. The activities of the project allowed us to collect useful data for the definition of measures for the management of outbreaks of salmonellosis in dairy cattle. The biosecurity and biocontainment measures adopted were effective in identifying shedders and reducing environmental contamination. Results at follow-up sampling demonstrated how the application of indirect prophylaxis measures drastically reduced the presence of *Salmonella* spp., leading to the extinction of the outbreak, thus constituting a possible model for the management of *Salmonella* spp. outbreaks in dairy herds, even in complex farm situations.

## Figures and Tables

**Table 1:** Sampling performed on the first farm visit.

Area of the Farm/Category	Tool	Number of Samples Collected
Calves	Rectal swabs	60
Milking cow area	Sponges	5
Milking cow area	Boots swabs	2
Dry cow area	Boots swabs	1
Youngstock area	Boots swabs	8
Bulk tank milk	-	1

**Table 2:** Results of sampling on the first farm visit.

Area of the Farm/Category	Tool	Number of Samples Collected	Positive Samples	Serotype Isolated
Calves	Rectal swabs	60	29 (6 out of 29 from symptomatic animals)	*S*. Bredeney
Milking cow area	Sponges	5	2	*S*. Bredeney
Milking cow area	Boot swabs	2	2	*S*. Bredeney
Dry cow area	Boot swabs	1	0	-
Youngstock area	Feces	8	1	*S*. Bredeney
Bulk tank milk		1	0	-

**Table 3:** Summary table listing the risks that emerged from the epidemiological investigations and the control measures suggested to address them.

Critical Points	Control Measures Suggested
No vehicle disinfection system at the entrance to the herd	Cleaning and disinfection procedure at the entrance
Rendering service for removal of carcasses passes near animals	Place a carcass stocking area outside the farm Avoid passage near area at risk
No all-in all-out practices	All-in all-out for calves housed in cages
No hospital cages for calves	Define an area as a hospital pen Keep positive animals separated from the others Workflow cleaning methods
Straw bedding material changed once a month	Remove bedding twice a month
Overcrowding	Reduce overcrowding
The same boots and cloths used in different barns	Use of disposable boots and gloves for different areas Separation of materials for different areas of the herd
Scarce hygiene practices	Detailed hygiene and disinfection protocol
Presence of synanthropic animals	The entrance of synanthropic was forbidden
Possible contact with wildlife	The entrance of wild animals was forbidden

## Data Availability

Data are contained within the article.
